# Microbial community metabolism: An annotated selection of World Wide Web sites relevant to the topics in environmental microbiology

**DOI:** 10.1111/1758-2229.13161

**Published:** 2023-05-05

**Authors:** Lawrence P. Wackett

**Affiliations:** ^1^ McKnight Professor, Department of Biochemistry, Molecular Biology & Biophysics BioTechnology Institute, University of Minnesota St. Paul MN 55108 USA

## Rich extracellular metabolomes

### https://www.nature.com/articles/s41564‐022‐01072‐5

This study analyzed amino acid auxotrophs in microbial communities from the Earth Microbiome Project and dataset of cooperating yeast communities.

## Community‐scale networks

### https://microbiomejournal.biomedcentral.com/articles/10.1186/s40168‐021‐01213‐8

This contribution describes a software tool called METABOLIC which uses annotated genomes, metabolic pathway analyses, and seeks to predict individual contributions to biochemical cycles.

## Models of community metabolism

### https://pubs.rsc.org/en/content/articlelanding/2021/mo/d0mo00154f

Models are as valuable as the material that goes into them. This paper discusses how to select the microbial community metabolism data that goes into models and available algorithms that use that data.

## Metabolic overlap

### https://www.frontiersin.org/articles/10.3389/fgene.2019.00989/full

This review starts with the question as to why in a community of one thousand microbes, and knowing many microbes have metabolism in common, that the microbes do not largely compete against each other. They found that metabolic overlap is highest in extreme environments and lowest in microbiomes found on or in animals, the built environment and soil.

## Predicting metabolic cooperation from genomes

### https://biologicalsciences.uchicago.edu/news/features/genomic‐structure‐microbial‐community‐metabolism

This writeup is about research into how the genomic structure of microbial communities can predict the overall metabolic framework.

## Predicting community dynamics via metabolism

### https://journals.asm.org/doi/10.1128/mSystems.00146‐19

There are many types of interactions in a microbial community. This paper posits that metabolic models serve well to predict various ecological interactions.

## Metabolic models of communities

### https://journals.asm.org/doi/full/10.1128/msystems.01270‐22?af=R

This study exams single genome‐based metabolic models and further uses nondynamic, steady‐state models to account for community function and growth.

## Chitin‐degrading consortium

### https://www.energy.gov/science/ber/articles/it‐isnt‐picky‐eaters‐drive‐soil‐microbial‐metabolism

Chitin can supply both carbon and nitrogen in support of microbial growth. Microbes that do not metabolize chitin were critical for the community studied here.

## Fitness advantages in microbial consortia

### https://journals.asm.org/doi/10.1128/msystems.00051‐22

This paper described a constructed community of two *E. coli* strains to study the metabolic dynamics of shared metabolites.

## Metabolic networks and smartgrids

### https://ieeexplore.ieee.org/document/9740437

Complex networks are common to cell metabolism and electronic circuits. This is a theoretical and computational study comparing those networks.

## Metabolomics databases

### https://www.metabolomicsworkbench.org/databases/externaldatabases.php

Metabolomics is one experimental tool for studying single cell and consortial metabolism. This site provides links to a list of metabolomics databases.

## Microbial pathway prediction system

### http://eawag-bbd.ethz.ch/predict/

This system predicts metabolic pathways linking reactions of key biodegradative and biocatalytic enzymes. Most of the pathways depicted by the prediction algorithm are based on enzymes from different microbes and thus represent a predicted consortial metabolism.

